# MAINTAINING WALKING THROUGH WALK ON!: A COMMUNITY-BASED WALKING PROGRAM FOR OLDER ADULTS

**DOI:** 10.1093/geroni/igad104.1290

**Published:** 2023-12-21

**Authors:** Barb Nicklas, Elizabeth Chmelo Kemp, Michelle Gordon, Jaime Hughes, Rahma Ajja, Justin Moore

**Affiliations:** Wake Forest School of Medicine, Winston Salem, North Carolina, United States; WFSM, Winston Salem, North Carolina, United States; WFSM, Winston Salem, North Carolina, United States; Wake Forest School of Medicine, Winston-Salem, North Carolina, United States; Wake Forest University School of Medicine – Winston-Salem, North Carolina, Winston Salem, North Carolina, United States; WFSM, Winston Salem, North Carolina, United States

## Abstract

The NIA-funded LIFE study showed that a walking-based intervention prevents mobility disability, even in those with compromised walking ability. However, increases in activity were not maintained following cessation of the intervention, suggesting novel approaches and accessible opportunities are needed to provide continued support for this population to maintain increased walking. We previously reported high feasibility and acceptability from a pilot of a supervised, group-based, and fee-for-service walking program (Walk On!) for adults with mobility challenges. The program focuses on walking stamina, and takes place two days/week for 12 weeks. Walk On! was offered for a total of ten consecutive sessions from late 2017 until March 2020 at a local church. The majority of attendees were >75 years (71%), female (65%), and had low levels of physical function (usual gait speed=0.79±0.16; 31% used an assistive device). Of the 49 unique (first-time) attendees who registered for Walk On!, only 18 (37%) did not re-register for at least one subsequent session. Thus, 63% (n=31) registered for at least 2 sessions, 51% (n=25) for at least 3 sessions, 20 (41%) for at least 4 sessions, and 15 (31%) for at least 5 of the 10 sessions. Mean attendance at scheduled group walking days for all attendees was 77%±21% for all attendees and did not decrease substantially for those attending multiple sessions. The safe, social, and accessible community-based Walk On! program may be one approach to sustain walking for older adults with mobility challenges. Future research will focus on measuring individual characteristics associated with maintenance.

